# Complete mitochondrial genome of *Copadichromis virginalis* (Cichlidae)

**DOI:** 10.1080/23802359.2016.1144106

**Published:** 2016-03-28

**Authors:** Jian Gao, Jia Zhao

**Affiliations:** BGI Education Center, University of Chinese Academy of Sciences, Shenzhen, China

**Keywords:** *Copadichromis virginalis*, mitogenome, phylogeny analysis

## Abstract

In this study, we report the complete mitochondrial genome of *Copadichromis virginalis* (Cichlidae fish). The mitochondrial genome is 16 704 bp in length and has a base composition of A (27.6%), T (30.1%), C (15.7%) and G (26.6%), demonstrating a slight bias of high AT content (57.6%). It contains 35 genes (13 protein-coding genes, 22 tRNAs and two rRNAs) and a non-coding A + T rich region (D-loop region). The mitochondial genome of *Copadichromis virginalis* presents a clear bias in nucleotied composition with a positive AT-skew and a negative GC-skew. Except for ND6 gene, all other protein-coding genes were located on the H-strand. *ND4* gene and *ND4L* gene were overlapped by 5 bp, *ATP8* gene and *ATP6* gene were overlapped by 9 bp. The nucleotide sequence of 13 protein-coding genes of *Copadichromis virginalis* and other 19 Cichlidae species were used for phylogenetic analysis. The result indicated *Copadichromis virginalis* a close relationship with species *Astatotilapia calliptera.*

With at least 2200 known species, cichlid fishes (Cichlidae), the freshwater-offshoot of the predominantly marine perciform suborder Labroidei, are among the most species-rich families of all teleost fishes (Fischer et al. 2013). The cichlid fishes inhabiting the Great Lakes of East Africa, Malawi, Tanganyika and Victoria have long been at the forefront of evolutionary research (Taylor & Verheyen 2001). Lake Malawi alone is inhabited by 500–1000 endemic species of haplochromine cichlid fishes (Turner 1994), which species are thought to have evolved from a single ancestral population in 1 million years (Taylor & Verheyen 2001). *Copadichromis virginalis*, belonging to Cichlidae family, are found mostly in open deep water in the intermediate habitat and keep plankton as major diet. No mitochondrial genome of this species was published before. So, in this study, we determined the mitochondrial genome of *C. virginalis* is and reconstructed phylogeny tree by ultilizing the whole mtDNA.

Adult *Copadichromis virginalis* (type species) were acquired originally from Lake Nyasa, Malawi (12˚0'5''S, 34˚30'7''E) through commercial suppliers. The muscle tissue was maintained in 96° alcohol after DNA extraction. The complete mitochondrial genome of *Copadichromis virginalis* (GenBank accession no. KU144677) was sequenced through Illumina sequencing method and assembled using SPAdes (Bankevich et al. 2012). The circular mitochondrial genome is 16 704 bp in length and has a base composition of A (27.6%), T (30.1%), C (15.7%), G (26.6%), demonstrating a slight bias of AT content (57.6%). It encoded one control region (D-loop region), two ribosomal RNA genes (12S and 16S rRNA genes), 13 protein-coding genes (PCGs) and 22 transfer RNA genes. The H-strand comprised two rRNA genes, 14 tRNA genes and 12 protein-coding genes, while the L-strand encompassed eight tRNA genes and one protein-coding gene (*ND6* gene). *ND4* gene and *ND4L* gene were overlapped by 4 bp, and *ATP8* gene and *ATP6* gene were overlapped by 45 bp. All coding genes use ATG as start codon with the exception of COX1 initiating with GTG. The small ribosomal rRNA(12S rRNA) has a length of 960 bp and the large ribosomal rRNA (16S rRNA) measures 1585 bp.

Phylogenetic analysis was implemented by utilizing shared 15 genes (12S rRNA, 16s rRNA, *ND1, ND2, COX1, COX2, ATP6, ATP8, COX3, ND3, ND4, ND4L, ND5, ND6* and *CYTB*) with other 21 closely related algae mitochondrial genomes (Figure 1). Multiply alignments of every single gene set of the 20 species were first done using MAFFT with default settings (Katoh et al. 2005), then were revised manually by Jalview 2.6.1 (Waterhouse et al. 2009). Finally, conserved regions were picked out by Gblocks 0.91b (Castresana 2000) to construct concatenated nucleotide sequences (10 143 bp of every single sequence). Phylogenetic tree constructed using RAxML version 8.1.12 (Stamatakis 2014). The relationships among the 20 taxa were well resolved with strong node values. *Copadichromis virginalis* was clustered into African cichlid group and exhibited a close genetic distance with *Astatotilapia calliptera*.

**Figure 1.**
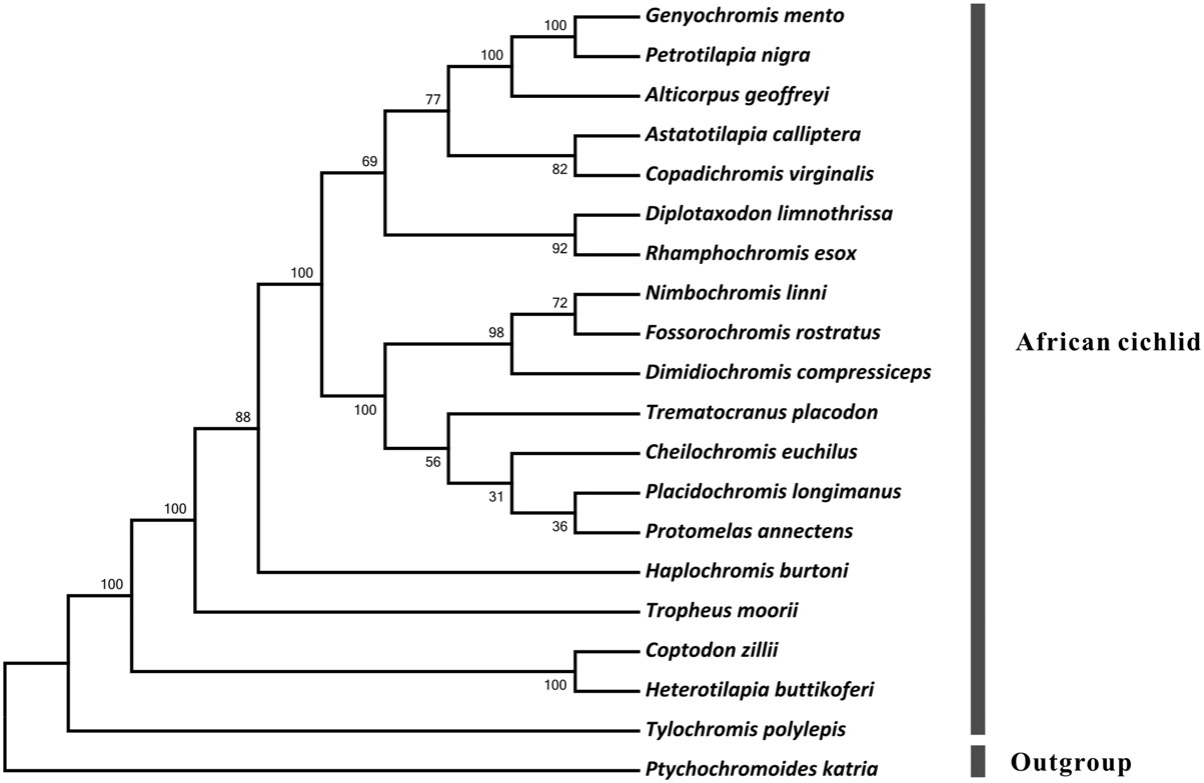
Phylogenetic relationships among Cichlidae spices. Numbers beside nodes are percents of 1000 bootstrap values. GenBank accession numbers of mitochondrial genomes used in this phylogeny analysis were listed: *Genyochromis mento* (JN628858); *Petrotilapia nigra* (JN628852); *Alticorpus geoffreyi* (KT277287); *Astatotilapia calliptera* (JN628855); *Diplotaxodon limnothrissa* (JN628851); *Rhamphochromis esox* (JN628860); *Nimbochromis linni* (JN628853); *Fossorochromis rostratus* (KT290557); *Dimidiochromis compressiceps* (JN628856); *Trematocranus placodon* (JN628850); *Cheilochromis euchilus* (JN252050); *Placidochromis longimanus* (KT309044); *Protomelas annectens* (KT188786); *Haplochromis burtoni* (KT221042); *Tropheus moorii* (HE961975); *Coptodon zillii* (KM658974); *Heterotilapia buttikoferi* (KF866133); *Tylochromis polylepis* (AP009509); *Ptychochromoides katria* (AP009507).
